# Alternative Behandlungsverfahren bei Vorhofflimmern: Was bringt Yoga?

**DOI:** 10.1007/s00399-022-00915-2

**Published:** 2022-12-29

**Authors:** Wilhelm Haverkamp, Wolf Sittner, Annika Haverkamp, Fabian Knebel

**Affiliations:** 1 Abteilung für Kardiologie und Metabolismus, Med. Klinik für Kardiologie, Campus Virchow-Klinikum, Charité – Universitätsmedizin Berlin, Augustenburger Platz 1, 13353 Berlin, Deutschland; 2 Klinik für Innere Medizin II: Schwerpunkt Kardiologie, Sana Klinikum Lichtenberg, Berlin, Deutschland; 3 Kardiologie im Spreebogen, Berlin, Deutschland; 4 Fakultät für Medizin, Universität Regensburg, Regensburg, Deutschland; 5 Medizinische Klinik mit Schwerpunkt Kardiologie, Campus Mitte, Charité – Universitätsmedizin Berlin, Berlin, Deutschland; 6 Berlin Institute of Health Center for Regenerative Therapies (BCRT), Berlin, Deutschland

**Keywords:** Vorhofflimmern, Risikofaktoren, Kardiovaskuläre Erkrankungen, Lebensstil, Lebensqualität, Atrial fibrillation, Risk factors for atrial fibrillation, Cardiovascular diseases, Lifestyle, Quality of life

## Abstract

Vorhofflimmern, die häufigste anhaltende Herzrhythmusstörung, ist mit einer erheblichen Morbidität, Mortalität und Inanspruchnahme von Gesundheitsleistungen verbunden. Vor dem Hintergrund, dass die zur Rhythmuskontrolle bei Vorhofflimmern eingesetzten Verfahren (Katheterablation, Antiarrhythmika) mit einer Reihe von Einschränkungen und Risiken behaftet sind, besteht ein wachsendes Interesse an erweiterten Behandlungsstrategien. Hierzu gehören eine Optimierung des Lebensstils, die Einstellung von Risikofaktoren für Vorhofflimmern und alternative Behandlungsverfahren, wie z. B. Yoga. Zu dessen Wirkung bei Vorhofflimmern liegen bislang nur wenige Studien vor. Diese sprechen aber dafür, das Yoga tatsächlich in der Lage sein dürfte, die Häufigkeit der Rhythmusstörung und ihre Progression zu vermindern. Auch die Risikofaktoren für Vorhofflimmern und die Lebensqualität werden positiv beeinflusst. Da unerwünschte Effekte und Komplikationen bei kompetenter Anleitung selten sind, kann regelmäßiges Yoga jetzt schon empfohlen werden. Um eindeutige, evidenzbasierte, praktische Empfehlungen geben zu können, sind aber weitere klinische Studien notwendig.

## Abstract (en)

Atrial fibrillation, the most common sustained cardiac arrhythmia, is associated with significant morbidity, mortality, and healthcare utilization. Since the procedures used to treat atrial fibrillation have a number of limitations and risks, there is a growing interest in alternative treatment strategies for patients with atrial fibrillation. One such option is yoga. To date, only a few studies are available on its effect on atrial fibrillation. However, these suggest that yoga may indeed be able to reduce the frequency of the arrhythmia and its progression. The risk factors for atrial fibrillation and quality of life in affected patients are also positively affected. As adverse effects and complications are extremely rare with competent guidance, yoga may already be recommended now. However, further clinical studies are needed to provide recommendations that meet evidence-based criteria.

Vorhofflimmern (VHF), die häufigste anhaltende Herzrhythmusstörung, ist mit einer erheblichen Morbidität, Mortalität und Inanspruchnahme von Gesundheitsleistungen verbunden. Es wird geschätzt, dass derzeit allein in Deutschland etwa 2,5 Mio. Menschen betroffen sind. Klassische Grundpfeiler der Behandlung sind die Frequenz- und Rhythmuskontrolle und die Antikoagulation [18]. Zu den Verfahren der Rhythmuskontrolle zählen die Therapie mit Antiarrhythmika und die in den letzten Jahren zunehmend häufiger eingesetzte Katheterablation. Für viele Patienten stellt die Katheterablation mittlerweile die Therapie der ersten Wahl dar. Beide Behandlungsverfahren sind allerdings mit einer Reihe von Einschränkungen hinsichtlich der Langzeitwirksamkeit und Risiken verbunden [18]. Aus diesem Grund besteht ein wachsendes Interesse an diese Verfahren ergänzende und in manchen Situationen sogar ersetzende Behandlungsstrategien [12, 15]. Zum einen geht es dabei um die Optimierung des Lebensstils und die Einstellung der Risikofaktoren für Vorhofflimmern, zum anderen um ergänzende und möglicherweise auch alternative Therapien wie Phytotherapeutika, Akupunktur und Verfahren der Mind-Body-Medizin, wie z. B. autogenes Training, Yoga, Tai-Chi und Qigong. Die Beurteilung der therapeutischen Wertigkeit der ergänzenden bzw. alternativen Verfahren wird vor allem dadurch erschwert, dass prospektive und kontrolliert durchgeführte Studien in ausreichender Größe gar nicht oder nur sehr eingeschränkt zur Verfügung stehen. Dies trifft auch für Yoga zu, dem zahlreiche positive Effekte auf die kardiovaskuläre Gesundheit zugeschrieben werden [13, 17]. Auch über eine positive Beeinflussung der Arrhythmieneigung (inkl. der Neigung zu Vorhofflimmern) wird berichtet [1, 32]. Die Verbreitung von Yoga nimmt weltweit kontinuierlich zu; nicht explizit aus medizinischen Gründen, sondern in erster Linie mit dem Ziel, hiermit die allgemeine körperliche und geistige Leistungsfähigkeit zu verbessern. Dies gilt auch für Deutschland. Eine 2018 durchgeführte repräsentative Umfrage des Berufsverbandes der Yogalehrer in Deutschland ergab, dass ca. 5 % der Deutschen Yoga praktizieren [8]. Der Anteil ist unter den Frauen deutlich höher als unter den Männern (9 % gegenüber 1 %). Es sind insbesondere Menschen mittleren Alters (25 bis 49 Jahre), die Yoga betreiben.

Vor diesem Hintergrund zielt die vorliegende Arbeit darauf ab, die verfügbaren Studiendaten zur Wirkung von Yoga bei Patienten mit Vorhofflimmern zusammenzufassen und den Einfluss von Yoga auf die Risikofaktoren von Vorhofflimmern zu diskutieren. Die Frage ist, inwieweit Yoga tatsächlich eine mögliche Therapieoption bei Vorhofflimmern darstellt, die einer intensiveren, stärker evidenzbasierten Überprüfung hinsichtlich ihrer Wirksamkeit bedarf. Die Übersicht beginnt mit einer kurzen Einführung in die Ziele von Yoga.

## Yoga – Grundlagen

Yoga ist eine sich in einer besonderen Denk- und Lebensweise ausdrückende Philosophie, die vor 2000 Jahren von Patanjali formuliert wurde [43]. Yoga strebt an, Körper, Geist und Seele in ein Gleichgewicht zu bringen. Ziel ist, den Geist zur Ruhe kommen zu lassen, d. h. ihn frei von inneren und äußeren Zwängen werden zu lassen. Es gibt zahlreiche Yogaformen. Die heute in Deutschland und anderen westlichen Ländern am häufigsten praktizierte Form von Yoga ist Hatha-Yoga, bei dem der Weg zur Selbsterfahrung körperliche Übungen (Asanas), Atemübungen (Pranayama) und Meditation beinhaltet [26]. Zentral sind die Asanas, bei denen es darum geht, den Körper durch Dehnung und Muskelanspannung bewusst zu steuern. Um im Yoga Fortschritte zu machen, sollte zwei- bis dreimal pro Woche geübt werden. Irgendwann geht es nicht mehr darum, zu üben, weil man muss (es sich zum Ziel gesetzt hat), sondern weil man sich körperlich, geistig und seelisch besser fühlt und übt, weil man es will.

Bei vielen Menschen, die heute Yoga praktizieren, stehen diese *höheren* Ziele nicht im Vordergrund. Ihnen geht es in erster Linie um eine bessere Körperbeherrschung und Fitness. Je älter Praktizierende werden, desto mehr scheinen präventive und medizinisch-therapeutische Effekte im Vordergrund zu stehen. Von einem Teil der Krankenkassen werden die Kosten für unter diesen Gesichtspunkten durchgeführtes Yoga ganz oder teilweise übernommen.

## Studien zur Wirkung von Yoga bei Vorhofflimmern

In Tab. 1 sind die zur Wirkung von Yoga bei Vorhofflimmern zur Verfügung stehenden Studien zusammengefasst. Zur direkten Wirkung von Yoga auf die Häufigkeit von Vorhofflimmern liegt lediglich eine Untersuchung vor. Es handelt sich um eine US-amerikanische, 2013 publizierte Studie bei Patienten mit symptomatischem paroxysmalem Vorhofflimmern [21]. Alle Studienteilnehmer unterzogen sich anfänglich einer 3‑monatigen nichtinterventionellen Beobachtungsphase. Anschließend absolvierten sie mindestens zweimal wöchentlich ein 60-minütiges Yoga-Training für die nächsten 3 Monate. Die Patienten bekamen eine DVD, mit deren Hilfe die Übungen zuhause durchgeführt werden konnten. Die Diagnostik erfolgte mit einem externen Ereignisrekorder. Die Häufigkeit von Vorhofflimmerepisoden während der Kontroll- und Studienphase wurden erfasst. Darüber wurden Daten zur Lebensqualität, zur selbsteingeschätzten Angst- und Depression erhoben. Yoga-Training führte zu einer signifikanten Reduktion der Häufigkeit symptomatischer und asymptomatischer Episoden von Vorhofflimmern (3,8 ± 3 vs. 2,1 ± 2,6, *p* < 0,001 bzw. 0,12 ± 0,44 vs. 0,04 ± 0,20; *p* < 0,001). Depressionen und Ängste nahmen ab (*p* < 0,001). Die Lebensqualität nahm signifikant zu (*p* < 0,001). Es ergab sich zudem eine signifikante Abnahme der Herzfrequenz und des systolischen und diastolischen Blutdrucks durch Yoga (*p* < 0,001).

**Table 1:** 

Autor, Publikationsjahr	Studiendesign	Gruppengröße (*n*)	Intervention	Assessments	Ergebnisse
Lakkireddy et al. 2013 [21]	Sequenzielles Design	52, 2 Phasen (Kontrolle, Yoga)	Kontrolle: Standardbehandlung Yoga: Standardbehandlung und 1 h Yoga zweimal pro Woche über 3 Monate	Episoden von Vorhofflimmern (symptomatisch, asymptomatisch); Lebensqualität, Angst und Depression (anhand von Fragebögen)	Yoga: signifikante Abnahme der Anzahl der symptomatischen und asymptomatischen Episoden von Vorhofflimmern; signifikant verbesserte Lebensqualität; signifikant reduzierte Angst und Depression (*p* < 0,05); signifikante Reduktion von Blutdruck und Herzfrequenz
Wahlström et al. 2017 [39]	Randomisierte, kontrollierte Studie	80 (Yoga, 40; Kontrolle, 40)	Kontrolle: Standardbehandlung Yoga: Standardbehandlung und 1 h Yoga pro Woche über 3 Monate	Blutdruck, Herzfrequenz und Lebensqualität (anhand von Fragebögen)	Yoga: signifikante Abnahme des systolischen und diastolischen Blutdrucks und der Herzfrequenz. Steigerung mehrerer Komponenten der Lebensqualität
Wahlström et al. 2020 [40]	Randomisierte, kontrollierte Studie	132 (Yoga, 44; Entspannung, 44; Kontrolle, 44)	Kontrolle: Standardbehandlung Entspannung: Standardbehandlung und Entspannungsübungen für 30 min einmal pro Woche über 3 Monate Yoga: Standardbehandlung und 1 h Yoga pro Woche über 3 Monate	Blutdruck, Herzfrequenz und Lebensqualität (anhand von Fragebögen)	Yoga: signifikante Abnahme des systolischen und diastolischen Blutdrucks; kein Einfluss auf die Herzfrequenz. Steigerung mehrerer Komponenten der Lebensqualität

Die beiden anderen in Tab. 1 aufgeführten Untersuchungen wurden von einer Arbeitsgruppe in Schweden durchgeführt. Im Vordergrund standen unterschiedliche Parameter der Lebensqualität. Eine Quantifizierung der Rhythmusstörung (z. B. eine Erhebung Häufigkeit von Arrhythmieepisoden) erfolgt nicht. Es wurde eine betont meditativ orientierte, aber auch aus Atem- und Körperübungen bestehende Yoga-Form eingesetzt. Einmal wöchentlich fanden Yoga-Sitzungen im Krankenhaus statt; die Patienten wurden angehalten, zusätzliche Sitzungen unter häuslichen Bedingungen (mit Hilfe einer mitgegebenen CD) durchzuführen.

Eine unter diesen Kautelen durchgeführte 12-wöchige randomisierte Pilotstudie bei 80 im Mittel knapp über 60 Jahre alten, mit einer Standardtherapie behandelten Patienten mit paroxysmalem Vorhofflimmern eingeschlossen wurden, ergab eine verbesserte Lebensqualität und eine Verringerung von Blutdruck und Herzfrequenz [39]. In die finale Studie wurden 132 Patienten mit symptomatischem Vorhofflimmern eingeschlossen [40]; es erfolge eine Randomisierung zu einer Kontrollgruppe ohne Intervention (*n* = 44), einer Yoga-Gruppe (*n* = 44) und einer Gruppe, die sich einmal wöchentlich einer 30-minütigen Sitzung unterzog, in der relaxierende Musik zur Entspannung gehört wurde. Die Standardtherapie blieb unbeeinflusst. Nach 12 Wochen gab es keine Unterschiede in der globalen gesundheitsbezogenen Lebensqualität zwischen den Gruppen. Yoga-Patienten zeigten allerdings im Verlauf in den Unterkategorien körperliche Schmerzen, allgemeine Gesundheit, soziale Funktion und psychische Gesundheit eine Verbesserung. In der Entspannungs- und Kontrollgruppe wurde keine Veränderung festgestellt. Der systolische und diastolische Blutdruck sank nur in der Yoga-Gruppe. Nach 12 Wochen gab es zwischen den Gruppen keine Unterschiede in der Herzfrequenz und dem NT-proBNP [40]. In einer Substudie wurden 12 Teilnehmer (jeweils 4 pro Gruppe) hinsichtlich ihres Befindens interviewt [39]. Patienten der Yoga-Gruppe gaben ein neues Gefühl der Existenz mit einem gesteigerten inneren Frieden und einer verbesserten Verbindung von Körper und Geist an. Dies entspricht dem Wesen von Yoga, dessen Ziel es ist, ein tieferes Verständnis des Ganzen und der Verbindungen zwischen Körper und Geist zu erlangen.

Die Autoren dieser Studien sind sich der Limitationen ihrer Studien (geringe Fallzahl etc.) bewusst. Es wird spekuliert, dass die positiven Effekte von Yoga bei paroxysmalem Vorhofflimmern durch eine Zunahme des parasympathischen Grundtonus (und reduzierten Fluktuationen des autonomen Nervensystems) und einer erhöhten Lebensqualität (u. a. durch eine verbesserte Stressbewältigung) mit der Folge einer positiven Wirkung auf das kardiale Remodeling bedingt sind [21, 40]. In keiner der Studien wurde über das Auftreten von unerwünschten Wirkungen bzw. Komplikationen berichtet.

## Einfluss von Yoga auf die Risikofaktoren für Vorhofflimmern

**Figure 1:**
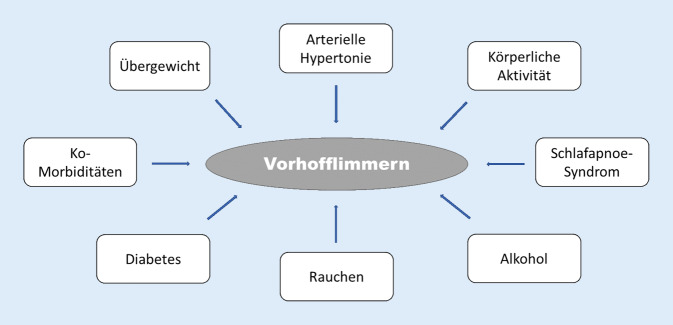

Bei der Entstehung von Vorhofflimmern spielen Risikofaktoren eine maßgebliche Rolle. Nichtmodifizierbare (Alter, Geschlecht, Genetik) können von modifizierbaren Risikofaktoren unterschieden werden [16, 22]. Abb. 1 zeigt eine Zusammenstellung der Risikofaktoren für Vorhofflimmern, die modifizierbar sind. Man darf annehmen, dass die Interaktion dieser Faktoren mit der individuellen Neigung zum Auftreten bzw. einer Progression der Rhythmusstörung komplex ist [22]. Die Fachgesellschaften empfehlen, diese Risikofaktoren möglichst optimal zu korrigieren bzw. zu behandeln [12, 18]. Zu nahezu allen modifizierbaren Risikofaktoren für Vorhofflimmern liegen Daten zum Einfluss von Yoga vor.

### Arterielle Hypertonie.

Bluthochdruck ist einer der wichtigsten Risikofaktoren für die Entwicklung von Vorhofflimmern. Das Vorhandensein von Bluthochdruck erhöht das Risiko für Vorhofflimmern um bis zu 50 % bei Männern und 40 % bei Frauen [7]. Bei Patienten mit bekanntem Vorhofflimmern liegt die Prävalenz von Bluthochdruck bei 60–80 % [27]. Durch eine konsequente Behandlung der arteriellen Hypertonie kann der Verlauf von Vorhofflimmern positiv beeinflusst werden [33].

Eine erst kürzlich publizierte Metaanalyse von 34 randomisierten und kontrollierten Studien zum Einfluss von Yoga auf den Blutdruck konnte eine signifikante Reduktion des systolischen und diastolischen Blutsdrucks nachweisen (mittlere Differenz: −6,49 mm Hg und −2,78 mm Hg; 95 % KI −8,94 bis −4,04 bzw. −4,11 bis −1,45; [28]). Fast alle Studien mit positivem Ausgang beinhalteten die Anwendung mehrerer Yoga-Komponenten (Asana, Pranayama und Entspannungsübungen) sowie ein regelmäßiges Praktizieren.

### Kardiale Komorbiditäten.

Strukturelle Herzerkrankungen sind wichtige Risikofaktoren für das Auftreten von Vorhofflimmern [18]. Für viele der hierunter fallenden Krankheitsbilder gilt, dass die Rhythmusstörung vor allem bei fortgeschrittener Krankheitsausprägung auftritt. Das Arrhythmierisiko nimmt weiter zu, wenn die Erkrankung von einer Herzinsuffizienz begleitet wird.

Sowohl bei chronischer koronarer Herzerkrankung [24] wie auch bei einer Herzinsuffizienz [35] zeigen aktuelle Metaanalysen unter Yoga eine gesteigerte Lebensqualität, eine bessere Einstellung von anderen kardiovaskulären Risikofaktoren und eine Abnahme der Anzahl kardiovaskulärer Ereignisse. Ein positiver Einfluss auf die Sterblichkeit zeigt sich nicht.

### Diabetes mellitus Typ 2.

Ein Diabetes mellitus Typ 2 und ein erhöhter Blutzuckerspiegel gehen mit einem signifikant erhöhten Risiko für die Entwicklung von Vorhofflimmern einher. Laut einer Metaanalyse von 32 Kohortenstudien mit mehr als 460.000 Diabetikern (und mehr als 10 Mio. nichtdiabetischen Kontrollpersonen) erhöht das Vorliegen eines Typ-2-Diabetes mellitus und das Risiko für Vorhofflimmern um 28 % (RR 1,28; 95 % KI 1,22–1,35; [5]). Bei Vorliegen eines Prädiabetes war das Risiko für Vorhofflimmern um 20 % erhöht. Es bestand eine lineare Beziehung zwischen steigenden Blutzuckerwerten und Vorhofflimmern. Ein Anstieg des Blutzuckerspiegels um 20 mg/dl (1,1 mmol/l) war mit einem um 1,11 (95 % KI 1,04–1,18) höheren VHF-Risiko verbunden [5].

Kurzfristig hat Yoga keinen wesentlichen Einfluss auf den Blutzucker bzw. den HbA1c-Wert, langfristig aber schon. Dies zeigt eine Metaanalyse von 4 Studien, die 2020 publiziert wurde. MDA, der HbA1c-Wert und die Nüchtern-Blutglukose nehmen unter Yoga signifikant ab [37]. Die diese Effekte vermittelnden Mechanismen scheinen, so wird diskutiert, vielfältig zu sein (Optimierung der Blutzuckerhomöostase, bessere Fitness, Gewichtsabnahme).

### Obstruktives Schlafapnoe-Syndrom.

Mehrere Beobachtungsstudien legen nahe, dass Patienten mit einem obstruktiven Schlafapnoe-Syndrom ein signifikant erhöhtes Risiko für Vorhofflimmern haben. In der Sleep Heart Health Study wurde festgestellt, dass das Risiko für Vorhofflimmern bei Patienten mit OSA um das Vierfache (95 % KI 1,03–15,7) höher war als bei Patienten ohne OSA [25]. In einer aktuellen Metaanalyse nahm das Risiko für das Auftreten von Vorhofflimmern für jeden Punkt, denn der AHI (Apnoe-Hypopnoe-Index) ansteigt, um 1,26 % zu (95 % KI 0,86–1,67 %; *p* < 0,05; [44]).

Dem Einfluss von Yoga auf den Schlaf wird in den letzten Jahren viel Aufmerksamkeit gewidmet. Kleinere Studien weisen darauf hin, dass sich Yoga zu einer konstruktiven Ergänzung in der Behandlung von Patienten mit Schlafapnoe darstellen könnte [19]. Yoga wirkt sich nicht nur direkt auf die oropharyngeale Muskulatur, die Atemmuster und nasale sowie respiratorische Pathologien aus, sondern hilft auch, die Risikofaktoren für Schlafapnoe (Fettleibigkeit, Bewegungsmangel etc.) positiv zu beeinflussen.

### Übergewicht.

Langfristige Nachbeobachtungen haben gezeigt, dass jede Einheit des Anstiegs des Body-Mass-Index (BMI) das Risiko für Vorhofflimmern um 3 % (95 % KI 1–5 %) erhöht [14]. Eine Metaanalyse von Studien zum Einfluss einer Gewichtsreduktion auf Vorhofflimmern zeigt, dass die Patienten, die ≥ 10 % ihres ursprünglichen Körpergewichts verloren hatten, nicht nur signifikant seltener Vorhofflimmern-Rezidive entwickeln (Risikoverhältnis: 0,29; 95 % KI 0,19–0,44), sondern auch hinsichtlich der Episodendauer und dem Schweregrad der Beschwerden profitierten; beide nahmen signifikant ab [2].

Zu den Wirkungen von Yoga auf das Körpergewicht liegen eine ganze Reihe von Studien vor [23]. Zusammenfassend lässt sich feststellen, dass Yoga als eine wirksame und sichere Maßnahme zur Verringerung des Body-Mass-Index bei übergewichtigen oder fettleibigen Personen angesehen werden kann.

### Körperliche Aktivität und Sport.

Dass regelmäßige körperliche Aktivität positive Auswirkungen auf das Herz-Kreislauf-System bzw. die Gesundheit hat, kann als gut belegt angesehen werden. Auch hinsichtlich der Neigung zu Vorhofflimmern ergeben sich positive Effekte. In einer Metaanalyse, in die 13 randomisierte Studien mit insgesamt 1155 Teilnehmern eingeschlossen wurden, reduzierte regelmäßiges Bewegungstraining das Wiederauftreten von Vorhofflimmern (relatives Risiko = 0,77: 95 % KI 0,60–0,99) und verbesserte die Lebensqualität [29]. Auf der anderen Seite ergeben sich Hinweise darauf, dass exzessiver Ausdauersport das Risiko für Vorhofflimmern zunehmen lässt [3].

Eine ganze Reihe von Studien zeigt auf, dass Yoga die gesundheitsbezogene Fitness verbessert, insbesondere die Muskelkraft und die kardiorespiratorische Fitness. Dies gilt nicht nur allgemein, sondern auch für Patienten mit Diabetes mellitus [42] und auch ältere Patienten [34].

### Alkohol.

Ein alkoholisches Standardgetränk (280–330 ml Bier, 150–180 ml Sekt, 30–40 ml Whisky oder hochprozentigem Schnaps, 60–80 ml Likör und 100–120 ml Rotwein) enthält etwa 10–12 g reinen Alkohol. Sieben solcher Getränke pro Woche (mäßiger Alkoholkonsum) erhöhten das Vorhofflimmern-Risiko [36]. Der Zusammenhang zwischen leichtem Alkoholkonsum (< 7 Standardgetränke pro Woche) und dem Risiko für Vorhofflimmern ist in den verfügbaren Studien weniger konsistent. Die klinische Erfahrung lehrt allerdings, dass es nicht selten Alkoholgenuss per se ist, der (unabhängig von der Alkoholmenge) bei Patienten mit bekanntem paroxysmale Vorhofflimmern Arrhythmieereignisse triggert. Damit scheint sich der mehrfach nachgewiesene kardioprotektive Effekt geringer Mengen von Alkohol nicht auf das Risiko für Vorhofflimmern zu erstrecken [38].

Studien deuten darauf hin, dass Yoga einen positiven Effekt auf Suchtverhalten (inkl. des Konsums von Alkohol) hat [41]. Das Praktizieren von Yoga verlangt einen klaren Kopf, so das sich feststellen lässt, Yoga und Alkohol passen nicht zueinander. Die Erfahrung zeigt, dass aktiv Yoga-Praktizierende häufig ihren Alkoholkonsum reduzieren bzw. auf alkoholfreie Alternativen (z. B. auch alkoholfreies Bier) umsteigen.

### Rauchen.

Eine kürzlich durchgeführte Metaanalyse von 29 Kohorten- und Fall-Kontroll-Studien zeigt ein erhöhtes Vorhofflimmerrisiko für aktuelle (relatives Risiko: 1,32; 95 % KI 1,12–1,56) und ehemalige Raucher (RR: 1,09; 95 % KI 1,00–1,18), wobei jede zusätzliche Zigarette, die pro Tag geraucht wird, mit einer 14 %igen Risikoerhöhung verbunden ist (RR: 1,14; 95 % KI, 1,10–1,20; [6]). Bei Rauchern mit Arrhythmieauslösern, die nicht aus der Pulmonalvenen stammen, wurde im Vergleich zu Nichtrauchern ebenfalls eine höhere Wahrscheinlichkeit eines Misserfolgs nach einer Ablation bei persistierendem Vorhofflimmern festgestellt [11].

Yoga führt bei Rauchern zu einer Verringerung des Verlangens nach Nikotin und einer verbesserten Hemmungskontrolle. Yoga wird als hilfreich bei der Entwöhnung von regelmäßigem Nikotinkonsum angesehen [10, 30].

## Diskussion

Wie für andere alternative Behandlungsverfahren gilt auch für Yoga, dass eine Bewertung schwierig ist, klinische Studien, die den Anforderungen der evidenzbasierten Medizin gerecht werden, fehlen. Die unzureichende Datenlage wird besonders deutlich, wenn es um Studien zur direkten Beeinflussung von Vorhofflimmern durch Yoga geht. Die einzige hierzu zur Verfügung stehende Studie hatte ein sequenzielles Design [21]. Einer 3‑monatigen Kontrollphase mit Standardbehandlung schloss sich eine ebenso lange Phase an, während der zusätzlich Yoga praktiziert wurde. Die Ergebnisse könnten durch einen Placeboeffekt von Yoga beeinflusst worden sein. Dieser Effekt ist bei Studien zu Yoga grundsätzlich nicht auszuschließen, da Scheinprozeduren verständlicherweise nicht möglich sind. Letztendlich erscheint bei der zusätzlichen Berücksichtigung der zahlreichen Untersuchungen zur Wirkung von Yoga auf die Risikofaktoren von Vorhofflimmern aber doch die Annahme erlaubt zu sein, dass sich auch bei den Kriterien der evidenzbasierten Medizin gerecht werdenden Studien ein positiver Behandlungseffekt nachweisen lassen sollte.

Aktuell sind eine ganze Reihe von Untersuchungen erschienen, die belegen, wie wichtig eine auch den Lebensstil und Risikofaktoren umfassende Behandlungsstrategie bei Vorhofflimmern ist. Erste Untersuchungen zeigen, dass die Rezidivrate der Katheterablation im Langzeitverlauf deutlich abnimmt, wenn diesem Vorgehen gefolgt wird [4]. Erst unlängst publizierte die American Heart Association ein 22-seitiges Konsensusdokument, dass die Optimierung des Lebensstils und Korrektur von Risikofaktoren zum vierten Grundpfeiler des Managements von Vorhofflimmern erhebt [12]. In der Praxis scheint hierauf aber eher selten geachtet zu werden. In den allermeisten Fällen wird, wohl basierend auf Studien [20], die darauf hinweisen, dass eine frühe Rhythmuskontrolle (Antiarrhythmika oder Katheterablation) im Verlauf von Vorhofflimmern besonders hilfreich ist, die Intervention ohne eine vorherige Optimierung des Lebensstils bzw. Einstellung von Risikofaktoren durchgeführt. Diese Studien vergleichen somit die therapeutische Intervention mit dem üblichen Vorgehen, das nicht die vorherige Optimierung des Lebensstils bzw. Einstellung von Risikofaktoren beinhaltet. Den Patienten selbst scheinen diese Zusammenhänge nur wenig bekannt zu sein; hat man sich zur Durchführung einer Intervention entschieden, dann wird auch von Patientenseite auf dessen Durchführung gedrängt. Auch Wartezeiten bleiben oft ungenutzt.

Bemerkenswert ist, dass es sich bei Yoga um eine Technik handelt, die, wenn sie von Fachkräften betreut und begleitet wird, arm an unerwünschten Wirkungen und Komplikationen ist. Ihre Durchführung kann relativ einfach an die Bedürfnisse und Möglichkeiten des Patienten angepasst werden. Auch Patienten mit Herzinsuffizienz profitieren [35]. Die Durchführung von Yoga wird zunehmend leichter gemacht. Bereits 2014 gab es in Deutschland über 6000 Yogastudios und -schulen [9]. Dabei steht Berlin mit über 300 Institutionen unangefochten an der Spitze. Für das Zuhausepraktizieren stehen CDs und DVDs zur Verfügung. Das Angebot zum Thema Yoga bei YouTube ist mittlerweile nahezu unüberschaubar geworden. Dass einzelne Anleitungen millionenfach angeklickt wurden, ist nicht selten. COVID-19 hat diesen Trend verstärkt. Nur am Rande sei bemerkt sei, dass mittlerweile für Yoga auch positive Effekte hinsichtlich COVID-19 nachgewiesen werden konnten. Yoga verfügt über das Potenzial, die zellvermittelte Immunität zu stärken; regelmäßig Yoga Praktizierende scheinen sich seltener zu infizieren und einen blanderen Verlauf bei einer Infektion zu haben [31].

### Implikationen für die weitere Forschung

Bevor Yoga als ein auf evidenzbasierten Kriterien als wirksam bewertetes, alternativ oder begleitend einsetzbares Behandlungsverfahren bei Vorhofflimmern empfohlen werden kann, sind weitere klinische Studien von ausreichender Qualität notwendig.

Geklärt werden muss, welche Form von Yoga am wirksamsten bzw. wirksamer als andere ist und ob meditationsbasierte Yogaformen ebenso wirksam oder sogar wirksamer sind als haltungsbasierte Interventionen. Unklar ist auch der Wert komplexer und dynamischer Yoga-Interventionen im Vergleich zu Yoga-Interventionen, die sich hauptsächlich auf die Körperhaltung konzentrieren.

Es ist davon auszugehen, dass auch die Effekte von Yoga einer positiven Dosis-Wirkungs-Beziehung folgen. Es dürfte Unterschiede in Abhängigkeit davon geben, wie häufig und wie intensiv praktiziert wird; ein einmaliges Praktizieren pro Woche sollte weniger wirksam als ein mehrmaliges oder gar tägliches Üben sein. Solche Studien fehlen bislang gänzlich.

Um die längerfristigen Auswirkungen von Yoga-Interventionen zu untersuchen, sind Langzeitstudien erforderlich, die die bislang übliche Dauer von 3 bis 6 Monaten deutlich überschreiten.

Eine große randomisierte Studie, die Yoga mit Untrainierten in Bezug auf harte kardiovaskuläre Endpunkte (wie etwa die Sterblichkeit, ggf. als Bestandteil eines kombinierten Endpunktes) in einer Langzeitbeobachtung vergleicht, wäre ebenso erforderlich und auch machbar. Ob eine solche Studie zukünftig zur Verfügung stehen wird, ist unklar. Es dürfte schwierig sein, hierfür Sponsoren zu finden.

## Fazit für die Praxis

- Yoga ist eine Mind-Body-Technik, die derzeit zunehmend Verbreitung findet.
- Erste Studiendaten deuten darauf hin, dass die Neigung zum Auftreten und einer Progression von Vorhofflimmern positiv beeinflusst werden kann.
- Weitere randomisierte und kontrollierte Studien sind notwendig, um den positiven Effekt abzusichern, seine Stärke besser einschätzen zu können und um die Mechanismen weiter aufzudecken, die den medizinischen Wirkungen von Yoga zugrunde liegen.
